# Factors associated with non-response to naldemedine for opioid-induced constipation in cancer patients: A subgroup analysis

**DOI:** 10.1371/journal.pone.0278823

**Published:** 2022-12-09

**Authors:** Yuko Kanbayashi, Mayumi Shimizu, Yuichi Ishizuka, Shohei Sawa, Katsushige Yabe, Mayako Uchida

**Affiliations:** 1 Faculty of Pharmacy, Department of Education and Research Center for Clinical Pharmacy, Osaka Medical and Pharmaceutical University, Takatsuki, Japan; 2 Department of Pharmacy, Seirei Hamamatsu General Hospital, Hamamatsu, Japan; 3 Department of Education and Research Center for Pharmacy Practice, Doshisha Women’s College of Liberal Arts, Kyotanabe, Japan; University of Tokyo, JAPAN

## Abstract

**Background:**

Opioid-induced constipation (OIC) is one of the most common adverse events of opioid therapy and can severely reduce quality of life (QOL). Naldemedine is the orally available peripheral-acting μ-opioid receptor antagonist approved for OIC treatment. However in daily clinical practice, some cancer patients show insufficient control of OIC even while receiving naldemedine.

**Objective:**

To identify factors associated with non-response to naldemedine in cancer patients.

**Methods:**

This study retrospectively analyzed 127 cancer patients prescribed naldemedine at Seirei Hamamatsu General Hospital in Japan between November 2016 and June 2021. For the regression analysis of factors associated with OIC, variables were extracted manually from electronic medical records. Naldemedine had been prescribed by the attending physician after the presence of OIC had been defined with reference to Rome IV diagnostic criteria. Naldemedine was evaluated as “effective” in cases where the number of defecations increased at least once in the first 3 days after starting naldemedine. Multivariate logistic regression analysis was performed to identify factors associated with non-response to naldemedine. The data used were from the group of patients who received naldemedine in our previous study.

**Results:**

Factors significantly associated with non-response to naldemedine included chemotherapy with taxanes within 1 month of evaluation of naldemedine effect (odds ratio [OR] = 0.063; 95% confidence interval [CI] = 0.007–0.568), and addition of or switching to naldemedine due to insufficient efficacy of prior laxatives (OR = 0.352, 95% CI = 0.129–0.966).

**Conclusion:**

The identification of factors associated with non-response to naldemedine prescribed for OIC may help improve QOL among cancer patients.

## Introduction

Opioid-induced constipation (OIC) is one of the most common adverse events of opioid therapy and can severely reduce quality of life (QOL) [1,2]. OIC is common among patients with advanced cancers, with a prevalence of approximately 51–87% among patients receiving opioids for pain management [1–4]. Naldemedine is the newest orally available peripheral-acting μ-opioid receptor antagonist (PAMORA) approved for OIC treatment in adult patients [5–7]. Naldemedine improves OIC by binding to opioid receptors in the gastrointestinal tract and antagonizing opioid analgesics [5]. The analgesic effects of many opioid analgesics are expressed mainly via the central μ-opioid receptor [1,2,5]. Naldemedine is a PAMORA designed so as not to inhibit the action of opioid analgesics in the central nervous system [5–7]. In phase III trials, naldemedine was more effective than placebo for increasing the frequency of spontaneous bowel movements among patients with OIC and cancer pain [6,7]. Naldemedine has also been shown to improve patient-rated constipation-related symptoms and QOL [6–8].

However, in daily clinical practice, some advanced cancer patients show insufficient OIC control even while receiving naldemedine. We showed that in our previous study, OIC was poorly controlled in 39.6% of patients, even when naldemedine was administered [9]. In addition, direct evidence remains lacking regarding the comparative efficacy of naldemedine compared with previous laxative treatments (osmotic laxative [e.g., magnesium and sulfate salts, lactulose and polyethylene glycol], stimulant laxative [e.g., anthranoid plant compounds, bisacodyl] or intestinal secretagogues [lubiprostone]) and PAMORAs (e.g., methylnaltrexone or naloxegol).

Previous studies have clarified that risk factors for constipation include female sex, advanced age, medications (e.g., anticholinergics, antipsychotics), diets, comorbidities, and mental states [10–12]. We also showed that the risk factors of OIC were high body mass index (BMI), chemotherapy including a taxane within 1 month of evaluation of laxative effect, no use of naldemedine, and addition or switching due to insufficient prior laxatives in advanced cancer patients [9]. Lavan et al. clarified in an observational study of an oncological population that constipation occurred in 20% of patients [13]. In that study, causative medications included systemic anticancer therapies (53.3%) and opioids (17.3%) [13]. Although a previous study clarified use of naldemedine within 2 days of opioid initiation was a predictor of efficacy of naldemedine, including patients with chronic pain, information remains limited for cancer patients [14,15]. This subgroup analysis of our previous study [9] was therefore undertaken to identify predictors of non-response to naldemedine prescribed for OIC among cancer patients, to help guide future strategies toward improving QOL in advanced cancer patients receiving pain treatment with opioids.

## Patients and methods

### Study period and participants

This subgroup analysis analyzed 127 cancer patients with cancer pain who were prescribed naldemedine at Seirei Hamamatsu General Hospital in Japan between November 2016 and June 2021. Patients who discontinued naldemedine within 2 days, were discharged within 2 days after starting naldemedine, or lacked data on defecation for the first 3 days after naldemedine prescription (missing data) were excluded. The data used were from the group of patients who received naldemedine in our previous study [9].

### Ethics and consent

All study protocols were approved by the Medical Ethics Review Committee at Seirei Hamamatsu General Hospital (approval no. 3310) and the Faculty of Pharmacy at Osaka Medical Pharmaceutical University (approval no. 0088). All procedures were performed in accordance with the ethical standards of Seirei Hamamatsu General Hospital, the Institutional Medical Ethics Review Committee at Osaka Medical Pharmaceutical University and the 1964 Declaration of Helsinki and its later amendments. Given the retrospective nature of this work, the need to obtain informed consent was waived for the individual participants included in the study, in accordance with the standards of the Seirei Hamamatsu General Hospital and Osaka Medical Pharmaceutical University of Medicine Institutional Medical Ethics Review Committee.

### Extraction of variables

Variables associated with potential risk factors for OIC were extracted from electronic medical records and used for regression analysis. Variables extracted were factors considered as potentially affecting OIC based on previous studies [1,9–13] or clinically rational mechanisms, including demographic data (age, BMI, body surface area [BSA]), clinical data (Eastern Cooperative Oncology Group performance status [ECOG-PS], and stage of cancer), medication-related data (chemotherapy administered within 1 month of the evaluation of naldemedine effect, anti-cancer drugs administered within 1 month of the evaluation of naldemedine effect, types of opioids, daily dose of opioid in milligrams of morphine-equivalents, concomitant laxative, concomitant use of the serotonin 5-hydroxytryptamine-3 (5-HT3) receptor antagonists, naldemedine prescription within 2 days of opioid initiation, and addition of or switching to naldemedine due to insufficient efficacy of prior laxatives), and cancer type.

### Outcomes measured

The presence of OIC was defined based on Rome IV diagnostic criteria [2] by the attending physician, who then prescribed naldemedine. Details of the Rome IV diagnostic criteria used for the diagnosis of OIC in this study have been published previously [16,17].

The retrospective nature of this study meant that insufficient data were able to be extracted regarding stool properties after prescribing naldemedine. Naldemedine was thus evaluated as “effective” in cases where the number of defecations increased at least once within the first 3 days after starting naldemedine.

### Statistical analysis

This study used logistic regression analysis, with the response *Y* being a binary categorical variable (naldemedine effective = 1; naldemedine not effective = 0) evaluated simultaneously with multiple predictors for OIC = *X*.

Independent variables were analyzed for multicollinearity (correlation coefficient |r| ≥ 0.7), since correlations among variables can lead to unreliable and unstable results of regression analyses. First, univariate logistic regression analyses between outcomes and each potential independent variable were performed. Subsequently, a multivariate logistic regression model was constructed by employing those candidate variables showing values of *P* < 0.20 in univariate regression or selected based on previous studies. Whether a difference in the effect of naldemedine was seen according to the concomitant use of opioids or laxatives was also analyzed. Wilcoxon/Kruskal–Wallis test was used to identify significant differences between groups.

For all statistical analyses, values of *P* < 0.05 (two-tailed) were considered significant. All analyses were conducted using JMP Pro® version 16.2 (SAS Institute, Cary, NC, USA).

## Results

**Fig 1:**
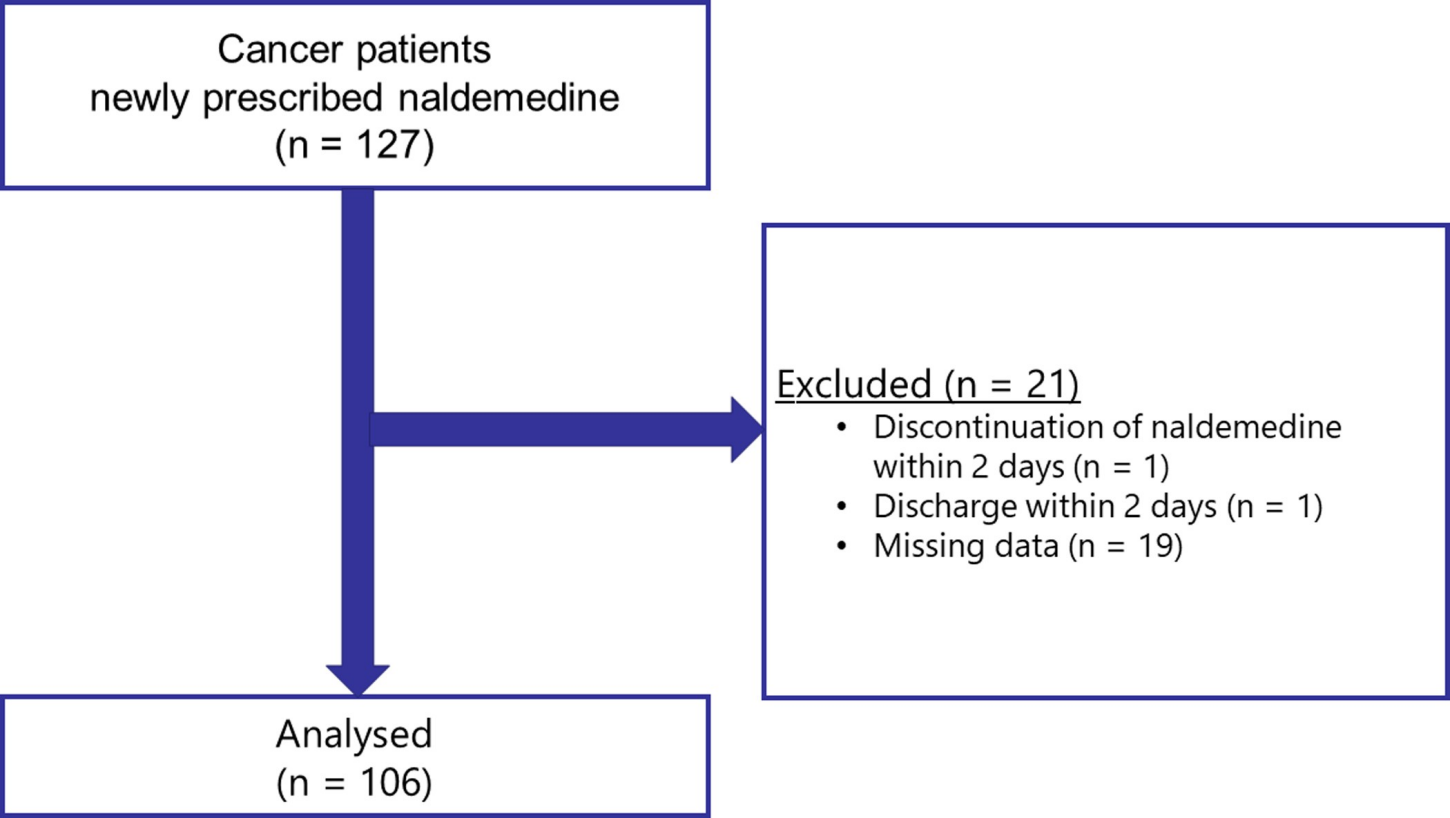
Of the 127 patients extracted, 106 patients were evaluated in this study. A total of 21 patients were excluded from this study due to discontinuation of naldemedine within 2 days (n = 1), discharge within 2 days (n = 1) or missing data (n = 19).

Of the 127 patients extracted, 106 patients were evaluated in this study (Fig 1). A total of 21 patients were excluded from this study due to discontinuation of naldemedine within 2 days (n = 1), discharge within 2 days (n = 1) or missing data (n = 19). Table 1 presents the clinical characteristics of the remaining 106 enrolled patients. Potential variables related to OIC and the results of univariate logistic regression analyses are shown in Table 2. The response rate to naldemedine was 60.4% (64/106). Univariate analyses only extracted “chemotherapy with taxane within 1 month of evaluation of naldemedine effect” as a significant factor.

**Table 1:** Patient demographics and baseline clinical characteristics.

	Patients (n = 106)
**Demographic data**	
Sex, male, n (%)	69 (65.9)
Age (y), median (range)	69 (42–89)
Height (cm), median (range)	162 (143–180)
Weight (kg), median (range)	51.9 (32.5–84)
Body mass index (kg/m^2^), median (range)	19.7 (13.0–28.9)
Body surface area (m^2^), median (range)	1.55 (1.22–2.04)
**Clinical data**	
ECOG PS status	
*ECOG PS status 0*, *n (%)*	0 (0)
*ECOG PS status 1*, *n (%)*	12 (11.3)
*ECOG PS status 2*, *n (%)*	46 (43.4)
*ECOG PS status 3*, *n (%)*	39 (36.8)
*ECOG PS status 4*, *n (%)*	9 (8.5)
Stage of cancer	
*Cancer stage I*, *n (%)*	0 (0)
*Cancer stage II*, *n (%)*	5 (4.7)
*Cancer stage III*, *n (%)*	5 (4.7)
*Cancer stage IV*, *n (%)*	96 (90.6)
**Cancer type**	
Colon, n (%)	10 (9.4)
Gastric, n (%)	5 (4.7)
Pancreatic, n (%)	28 (26.4)
Esophageal, n (%)	7 (6.6)
Lung, n (%)	16 (15.1)
Breast, n (%)	3 (2.8)
Head and neck, n (%)	19 (17.9)
Urologic, n (%)	8 (7.6)

ECOG PS, Eastern Cooperative Oncology Group performance status.

**Table 2:** Extracted variables, and results of univariate logistic regression analyses (n = 106).

	Patients (n = 106)	Naldemedine not effective (n = 42)	Naldemedine effective (n = 64)	Odds ratio (95%CI)	*P* value*
**Demographic data**					
Sex, male, n (%)	69 (65.9)	30 (71.4)	39 (60.9)	0.62 (0.27–1.44)	0.269
Age (y), median (range)	69 (42–89)	69 (42–88)	70 (42–89)	1.01 (0.97–1.04)	0.777
Body mass index (kg/m^2^), median (range)	19.7 (13.0–28.9)	19.7 (13.0–28.9)	19.6 (14.2–28.8)	0.95 (0.84–1.08)	0.434
Body surface area (m^2^), median (range)	1.55 (1.22–2.04)	1.58 (1.22–1.84)	1.55 (1.24–2.04)	0.30 (0.02–4.06)	0.365
**Clinical data**					
Eastern Cooperative Oncology Group performance status (0/1/2/3/4), n (%)	0/12/46/39/9 (0/11.3/43.4/36.8/8.5)	0/4/21/15/2 (0/9.5/50/35.7/4.8)	0/8/25/24/7 (0/12.5/39.1/37.5/10.9)	1.19 (0.73–1.94)	_
Eastern Cooperative Oncology Group performance status 3 & 4, n (%)	48 (45.3)	17 (40.5)	31 (48.4)	2.46 (0.48–12.4)	0.278
Stage of cancer (I/II/III/IV), n (%)	0/5/5/96 (0/4.7/4.7/90.6)	0/3/0/39 (0/7.1/0/92.9)	0/2/5/57 (0/3.1/7.8/89.1)	1.01 (0.37–3.33)	-
Cancer Stage IV, n (%)	96 (90.6)	39 (92.9)	57 (89.1)	0.63 (0.15–2.57)	0.516
**Medication-related data**					
Chemotherapy given within 1 month of evaluation of naldemedine effect, n (%)	42 (39.6)	19 (45.2)	23 (35.9)	0.68 (0.31–1.50)	0.339
Concomitant use of serotonin 5-HT3 receptor antagonist	33 (31.1)	15 (35.7)	18 (28.1)	0.70 (0.31–1.62)	0.410
Anti-cancer drugs administered within 1 month of the evaluation of naldemedine effect					
*Taxanes*, *n (%)*	9 (8.5)	7 (16.7)	2 (3.1)	0.16 (0.03–0.82)	**0.028**
*Fluorouracil*, *n (%)*	15 (14.2)	5 (11.9)	10 (15.6)	1.37 (0.43–4.34)	0.592
*Platinum*, *n (%)*	24 (22.6)	9 (21.4)	15 (23.4)	1.12 (0.44–2.86)	0.809
Types of opioids					
*Morphine*, *n (%)*	15 (14.2)	4 (9.5)	11 (17.2)	1.00 (1.00–1.01)	0.606
*Fentanyl*, *n (%)*	5 (4.7)	2 (4.8)	3 (4.7)	0.98 (0.16–6.15)	0.986
*Oxycodone*, *n (%)*	73 (68.9)	31 (73.8)	42 (65.6)	0.68 (0.29–1.60)	0.375
Daily dose, morphine-equivalents (mg), median (range)	40 (10–576)	45 (10–144)	40 (15–576)	1.00 (1.00–1.01)	0.606
Concomitant laxative					
*Magnesium oxide*, *n (%)*	18 (17.0)	5 (11.9)	13 (20.3)	1.89 (0.61–5.75)	0.265
*Sennoside*, *n (%)*	8 (7.6)	2 (4.8)	6 (9.4)	2.07 (0.39–10.8)	0.388
*Lubiprostone*, *n (%)*	3 (2.8)	1 (2.4)	2 (3.1)	1.32 (0.12–15.1)	0.822
Naldemedine prescription within 2 days of opioid initiation, n (%)	10 (9.4)	4 (9.5)	6 (9.4)	0.98 (0.26–3.71)	0.980
Addition of or switching to naldemedine due to insufficient prior laxative, n (%)	63 (59.4)	29 (69.0)	34 (53.1)	0.51 (0.22–1.15)	0.105
**Cancer type**					
Colon, n (%)	10 (9.4)	3 (7.1)	7 (10.9)	1.60 (0.39–6.56)	0.516
Gastric, n (%)	5 (4.7)	1 (2.4)	4 (6.3)	2.73 (0.29–25.3)	0.376
Pancreatic, n (%)	28 (26.4)	13 (31.0)	15 (23.4)	0.68 (0.29–1.64)	0.392
Esophageal, n (%)	7 (6.6)	3 (7.1)	4 (6.3)	0.86 (0.18–4.08)	0.856
Lung, n (%)	16 (15.1)	6 (14.3)	10 (15.6)	1.11 (0.37–3.33)	0.851
Breast, n (%)	3 (2.8)	0	3 (4.7)	-	-
Head and neck, n (%)	19 (17.9)	9	10 (15.6)	0.69 (0.25–1.88)	0.47
Urologic, n (%)	8 (7.6)	4 (9.5)	4 (6.3)	0.63 (0.15–2.68)	0.54

CI, confidence interval; 5-HT3, 5-hydroxytryptamine-3.

* *P* value pulled from univariate logistic regression analyses.

Multicollinearity was detected in BMI and BSA. BMI, which was considered the most clinically relevant for constipation, was used for analysis. The selection procedure using factors showing values of *P* < 0.20 in univariate regression or selection based on previous studies identified the following variables: BMI, chemotherapy with taxane within 1 month of evaluation of naldemedine effect, daily dose of opioid, use of naldemedine within 2 days of opioid initiation, and addition of or switching to naldemedine due to insufficient efficacy of prior laxatives. Neither platinum-based nor fluorouracil-based protocols other than taxanes were extracted as significant factors in the univariate analysis.

Multivariate logistic regression analysis was performed using these variables. Significant factors identified included chemotherapy with taxanes within 1 month of evaluation of naldemedine effect (odds ratio [OR] = 0.063; 95% confidence interval [CI] = 0.007–0.568), and addition of or switching to naldemedine due to insufficient efficacy of prior laxatives (OR = 0.352, 95% CI = 0.129–0.966) (Table 3). Since the ORs were less than 1.0, both factors were extracted as factors associated with non-response to naldemedine.

**Table 3:** Relative odds of naldemedine effectiveness in a multivariate model (n = 106).

Variable	Odds ratio	95% confidence interval		*P* value
		Lower endpoint	Upper endpoint	
Body mass index	0.890	0.776	1.022	0.098
Taxanes	0.063	0.007	0.568	**0.014**
Daily dose in morphine-equivalents (mg)	1.002	0.995	1.009	0.585
Naldemedine prescription within 2 days of opioid initiation	0.411	0.085	1.981	0.268
Addition of or switching to naldemedine due to insufficient prior laxative	0.352	0.129	0.966	**0.043**

No differences were evident in the effect of naldemedine among types of concomitant opioids (*P* = 0.658) or according to the concomitant use of laxatives (*P* = 0.536).

## Discussion

The results of this study showed that OIC was poorly controlled when taxane chemotherapy was administered within 1 month of evaluation of the naldemedine effect or with addition of or switching to naldemedine due to insufficient efficacy of prior laxatives. Our results are similar to those of our previous study [9], suggesting that patients prone to developing OIC may also be non-responsive to naldemedine. This study also showed that the effect of naldemedine was not different types of concomitant opioids or according to the concomitant use of laxatives.

Taxanes are anticancer drugs that have an adverse effect of constipation due to neuropathy [18]. Taxane-induced neuropathy persists for a prolonged period in clinical practice. If taxanes were administered within 1 month of evaluation of naldemedine effect, those adverse effects may still be present. As naldemedine is a PAMORA, control of constipation caused by taxanes may be difficult. Taxane-induced neuropathy may also affect the enteric nervous system and may decrease intestinal motility. Colonic irritant laxatives such as castor oil may thus be preferable. If paralysis of the intestinal tract occurs, intestinal decompression or other measures may be necessary. This point needs further validation. On the other hand, naldemedine is primarily metabolized by cytochrome P450 3A4 (CYP3A4) to form nor-naldemedine [19]. A previous study showed that coadministration of a P-glycoprotein inhibitor, CYP3A inhibitors, and a CYP3A inducer had notable effects on the pharmacokinetics of naldemedine [19]. In this study, some patients were receiving taxane chemotherapy at the time of evaluation of naldemedine effect. Enzyme systems such as CYP3A4 participate in metabolism of taxanes such as paclitaxel and docetaxel [20,21]. Although no reports have described drug-drug interactions between naldemedine and taxanes, the possibility of reduced naldemedine effects due to drug-drug interactions needs to be kept in mind, particularly in patients undergoing chemotherapy with taxanes. This point needs further verification using data from daily clinical practice. In recent years, new opioid prescriptions are often made by oncologists during chemotherapy. Thus, this information is useful for physicians who examine patients with breast, ovarian, or lung cancers who are often prescribed taxanes, and may help to improve QOL for patients. In particular, since these cancer patients are often treated on an outpatient basis, patients need to receive explanations that they should carefully monitor their own bowel movements at home. This information will also prove useful for pharmacists in providing medication guidance. However, the number of patients treated with taxanes in this study was small (9 patients). Therefore, further studies with larger numbers of patients are needed to establish evidence.

Addition of or switching due to insufficient prior laxatives was also extracted as a factor associated with non-response to naldemedine being prescribed for OIC. Naldemedine is a PAMORA, and the mechanism of action suggests that efficacy may be greater for a shorter duration of opioid exposure. Administration of naldemedine from the initiation of opioids may also reduce the risk of developing the opioid-withdrawal symptoms that can be seen when naldemedine is started during opioid administration [22,23]. On the other hand, the factor “naldemedine prescription within 2 days of opioid initiation” was not extracted as a significant factor in the present study. Some patients were undergoing chemotherapy at the time of evaluation of naldemedine effect. Recently, the serotonin 5-HT3 receptor antagonists have been commonly used in preventing chemotherapy-induced nausea and vomiting. However, the serotonin 5-HT3 receptor antagonist may have also acted on serotonin receptors in the intestine and decreased intestinal motility [24]. Thus, laxative therapy is often indicated. Although not significant in this study, it has been suggested that concomitant use of the serotonin 5-HT3 receptor antagonists is a nonresponding factor of naldemedine. The guidelines for OIC also suggest that classic laxatives should be used first, with the use of novel constipation treatments or PAMORA recommended only if the effects prove insufficient [25,26]. The appropriate timing of naldemedine administration needs to be verified in the future.

Although BMI was not a significant factor, our results showed that naldemedine tended to be ineffective for patients with higher BMI. Previous studies have reported obesity as a risk factor for constipation [27]. The results of this study were thus consistent with previous findings, and further study of this issue is warranted.

Daily dose of opioid was also not extracted as a significant factor. Roeland et al. showed a weak to absent association between total daily opioid dose and constipation in patients with cancer [28]. This result was again consistent with our findings. Our results suggest that OIC can be controlled with naldemedine regardless of the opioid dose.

OIC has a considerable effect on QOL for cancer patients receiving opioids. Cancer patients may occasionally discontinue opioid use due to OIC and endure pain. Patients with advanced cancer are also likely to experience severe distress, reduced work productivity, poor QOL, and increased healthcare utilization [1,29]. Several studies have reported improved QOL for cancer patients with improvements in constipation [30,31]. Clinicians should thus be alert to the occurrence of OIC and work to improve this symptom. The identification of factors associated with non-response to naldemedine being administered for OIC in this study may therefore help improve QOL for cancer patients.

In the present study, no difference in the effect of naldemedine was seen among types of concomitantly used opioids or laxatives. Among opioids, fentanyl has been reported to have a lower risk of constipation as a side effect [32]. However, the type of concomitantly used opioids in this study did not appear to affect the efficacy of naldemedine. Concomitant use of laxatives was also not found to have any effect on the efficacy of naldemedine.

Several limitations to the current study need to be considered. First, the retrospective nature of the study may have decreased the validity of the data obtained. That is, potential confounding, selection, and information biases could not be fully excluded in this study. Second, completely controlling for factors affecting constipation was difficult, given the wide variety of such factors, including medications (e.g., anticholinergics and antipsychotics), diet, comorbidities (e.g., cerebrovascular diseases), and mental state. Third, since this study was performed at a single institute, and it only examined a relatively small number of patients, some degree of selection bias would have been present. The present findings therefore need to be confirmed in further studies. Nevertheless, these results may help improve QOL among patients with advanced cancer and cancer pain.

In conclusion, administration of a chemotherapeutic regimen that included taxanes within one month before the evaluation of naldemedine effect and addition of or switching to naldemedine due to insufficient efficacy of prior laxatives were identified as factors associated with non-response to naldemedine being administered for OIC in cancer patients.
